# Effect of endodontic treatment on periodontal healing of grade 3 endo-periodontal lesions without root damage in periodontally compromised patients—a retrospective pilot study

**DOI:** 10.1007/s00784-020-03560-6

**Published:** 2020-09-19

**Authors:** Maurice Ruetters, Ti-Sun Kim, Johannes Krisam, Shirin El-Sayed, Nihad ElSayed

**Affiliations:** 1 Section of Periodontology, Clinic for Conservative Dentistry, University Hospital Heidelberg, Im Neuenheimer Feld 400, 69120 Heidelberg, Germany; 2 Institute of Medical Biometry and Informatics, University Hospital Heidelberg, Im Neuenheimer Feld 130.3, 69120 Heidelberg, Germany

**Keywords:** Periodontal disease, Endo-periodontal lesions, Periodontal attachment loss

## Abstract

**Objectives:**

There is little evidence about the effect of different treatment protocols for grade 3 endo-periodontal lesions without root damage in patients with periodontitis according to the new classification of periodontal disease. The aim of this study is to evaluate the impact of endodontic treatment on the achievement of periodontal healing.

**Materials and methods:**

Teeth with the initial diagnosis endo-periodontal lesion without root damage grade 3, treated with a standardized endodontic treatment protocol, were included in this study. A retrospective analysis was performed to assess the impact on periodontal healing by evaluating probing pocket depth (PPD), clinical attachment gain (CAL), and periapical index score (PAI).

**Results:**

Nineteen teeth and 13 patients were included. A mean reduction of 3.19 ± 3.41 mm in PPD was recorded. The mean CAL gain was 2.33± 3.75 mm. Five teeth (45.4%) showed an improvement of PAI and were classified as treatment success.

**Conclusions:**

The results failed to show a highly predictable treatment outcome for endo-periodontal lesion grade 3 without root damage in patients with periodontitis. However, endodontic therapy alone resulted in treatment success for some of the teeth, which would otherwise have had a poor prognosis.

**Clinical relevance:**

Endo-periodontal lesions can often be challenging for dentists in daily clinical practice. To date, there is not much evidence for practitioners to rely on. Therefore, this study aims to strengthen the evidence for the management and treatment of endo-periodontal lesions. Although the outcome is not highly predictable yet, teeth with the initial diagnosis endo-periodontal lesion without root damage grade 3 can benefit from an endodontic treatment.

## Introduction

Combined periodontal endodontic lesions can be of different origins. They can either be of endodontic origin, of periodontal origin, or of both. There are different kinds of pathways that might cause such a lesion, anatomical pathways and non-physiological pathways. Anatomical pathways connecting the tissues are the apical foramina and the branches of the root canal system, also named accessory canals, which can communicate with the periodontal ligament. Most of them are located in the apical third [1]. Another possible pathway are the dentine tubules, which can mostly be found at the cervical region [2]. Non-physiological pathways can be created by iatrogenic perforations and vertical root fractures for example. Similarities of the endodontic bacterial flora and the flora of periodontal pockets suggest that cross-infections are possible. However, research showed that the flora of periodontal ligament appears to be much more complex than the flora of the root canal [3]. Primary endodontic lesions can cause secondary periodontal lesions. An infected pulp will always lead to a periapical reaction if no root canal treatment is performed [4]. It cannot always be forecasted if this reaction leads to a periodontal lesion, as this depends on the individual situation. Reactions can also occur at accessory root canals in the furcation or the middle part of the root. Sometimes these lesions mimic early stages of periodontitis.

Whether primary periodontal lesions cause secondary endodontic lesions is subject to discussion. It is known that a pulpal involvement can happen if a periodontal lesion reaches the apex of a tooth. However, a marginally located periodontitis is unlikely to cause endodontic inflammation through the infection of dentine tubules [5]. One theory states that this is almost not possible due to the outward directed flow in the dentinal tubules of vital teeth [6]. If the teeth are still vital and the defect is solely of periodontal nature, root canal treatment should be avoided. It is important to note that a sensitivity response to a cold test represents vitality in 90%, and to an electric test in 84% [7]. Moreover, partial pulp necrosis is common in multi-rooted teeth. Therefore, one cannot always assume vitality of the pulp by sensitivity testing.

Combined lesions are hard to tackle. Once an endodontic and periodontal lesion communicate, a true combined lesion exists. It is currently recommended to first treat the endodontic lesion with a root canal treatment, and wait for 3 months before reevaluation. If the lesion still persists, an additional periodontal treatment of the lesion may be necessary. This treatment recommendation is based on the assumption that infected root canal areas can be sealed and prior scaling may inhibit reattachment [8].

Several classification systems have been established to categorize those lesions. The most widely known might be the one of Simon and Glick [9]. He classified five different types of lesions from class I to V. More recently the new classification by workgroup 2 of the 2017 World Workshop on the Classification of Periodontal and Peri-Implant Diseases and Conditions was published [10]. In this classification, combined lesions were categorized into two groups: those teeth with damage of the roots and those without damage of the roots. The three subgroups of those with damage of the roots are root fractures, perforation of the roots, and external root resorptions. Those without damage of the roots are divided into two subgroups of which each has three different severity codes. The first subgroup includes combined lesions of patients with periodontitis. The second subgroup represents combined lesions of patients without periodontitis. The severity codes are grades 1, 2, and 3 and give information of the anatomical morphology of the defect. Grade 1 includes narrow, deep pockets at one site of a tooth, while grade 2 includes wide, deep pockets at one site of a tooth and grade 3 includes deep pockets at more than one side of a tooth [10].

To date, there is little evidence about the effect of different treatment protocols for true combined endo-periodontal lesions, especially not for the new classification. Therefore, this study aims to evaluate the effect of sole endodontic treatment of teeth diagnosed with combined endo-periodontal lesions without root damage grade 3 in periodontally compromised patients on periodontal healing.

## Material and methods

The hypothesis was that sole endodontic treatment had facilitated periodontal healing. The primary outcome measure was the change of pocket probing depth, and secondary outcomes were change of PAI (periodontal apical index) and change of attachment loss (Tables 1 and 2).

**Table 1:** Quality score proposed by Farzaneh et al. 2004 [11]

	Description
Length	
Adequate	Filling 0–2 mm short of the root end
Short	Filling 2 mm short of the root end
Long	Filling of the root end
Density	
Good	Homogenous appearance, without voids
Poor	Detectable voids
Missed canal	≥ 1 canal not filled

**Table 2:** PAI score by Orstavik [12]

Score	Description
1	Normal periapical structures
2	Small change in bone structure
3	Changes in bone structure with some mineral loss
4	Periodontitis with well-defined radiolucent area
5	Severe periodontitis with exacerbating features

Nineteen teeth with the initial diagnosis combined endo-periodontal lesions without root damage grade 3 from 13 periodontally compromised patients were included in the study. All teeth were treated with a standardized endodontic treatment protocol and were analyzed retrospectively. All patients underwent a systematic periodontitis therapy which included a full mouth disinfection and, if necessary, adjunctive antibiotic therapy according to van Winkelhoff (375 mg amoxicillin and 250 mg metronidazole, three times daily for a period of 7 days) [13, 14]. The affected teeth with combined endo-periodontal lesion were exempted from subgingival debridement. They had never been instrumented subgingivally during the study interval. All endodontic treatments were performed at the Department of Restorative Dentistry at the University Hospital Heidelberg.

The study was approved by the local ethics committee (S-312/2020).

### Endodontic treatment protocol

All treatments were performed with the application of rubber dam. Instrumentation was performed with hand and rotary files (Reciproc®). All root canals were irrigated intermittently with ultrasonically activated sodium hypochlorite (3%) and chlorhexidine (2%). To avoid the formation of precipitates of parachloraniline in between the sodium hypochlorite and chlorhexidine irrigations, the canals were rinsed with ethyl alcohol. At the end of the first appointment, calcium hydroxide powder was mixed with chlorhexidine to form a pasty consistency, and the paste was applied into all canals. A second appointment was set at least 2 weeks later for obturation of the root canals. The obturation was performed with either thermoplastic filling or lateral condensing techniques using gutta-percha.

Periapical radiographs were taken before, during, and 6 to 17 months after endodontic treatment.

### Clinical examination

All patients received a dental checkup and a periodontal assessment recording PPD, CAL, furcation involvement, and tooth mobility before and after active periodontal treatment. The probing of the teeth with combined endo-periodontal lesions was performed at reevaluation 6 months after endodontic treatment at the earliest. Further assessment of periodontal parameters was made at the appointments of supportive periodontal care, with intervals adjusted at individual risk. For a better correlation of results to clinical practice, teeth showing improvement of PPD and CAL of ≥ 50% were classified as “success,” others as “failure.”

Restorations, caries, and sensitivity of the included teeth before endodontic treatment were recorded as well as smoking status and antibiotic therapy during full mouth disinfection.

### Radiographic examination

Periapical radiographs were taken before treatment and earliest 6 months after beginning of the endodontic treatment using parallel technique to ensure high reproducibility. The PAI was analyzed at the apex of the root in each periapical radiograph by one calibrated investigator blinded to the clinical outcome [12]. The characterization of the PAI is shown in Table 2. It consists of a 5-point scale and is designed to evaluate the apical situation of a tooth. Five radiographs served as a reference. The scores correlate with confirmed histology. It was used to quantify radiographic changes only at the apex of the treated teeth.

The quality of the root canal filling was rated radiographically according to a scheme used by Farzaneh et al. [11]. Two investigators (MR and SE), both with 8 years of experience in clinical and radiographic diagnostics, rated independently of each other. If ratings differed, a consensus was made. Length and density were rated separately. A precise characterization of the rating scores is shown in Table 1.

**Fig. 1:**
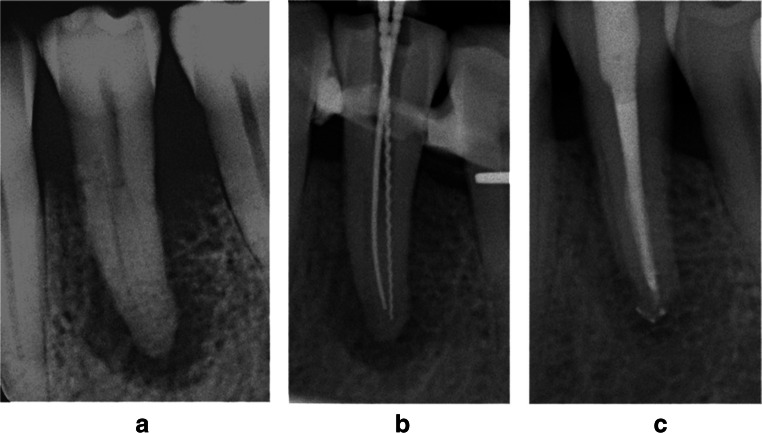
Tooth 35 (FDI) with combined endo-periodontal lesion of a patient with periodontitis grade 3 with 2 root canals. **a** Before treatment, PAI score 5, PPD 9 mm buccal and distobuccal. **b** Measuring of length. **c** Situation 15 months after treatment, PAI score 2, PPD 2 mm buccal and 2 mm distobuccal

The software application used was Sidexis Next Generation 1.53 (Sirona dental systems, Bensheim, Germany). Radiographic evaluations were all performed on a certified monitor (EIZO, RadiForce RS 210) in the same dark room. Teeth with improvement of the PAI score were classified as “successful,” others as “failure.” Fig. 1.

### Statistical analysis

**Fig. 2:**
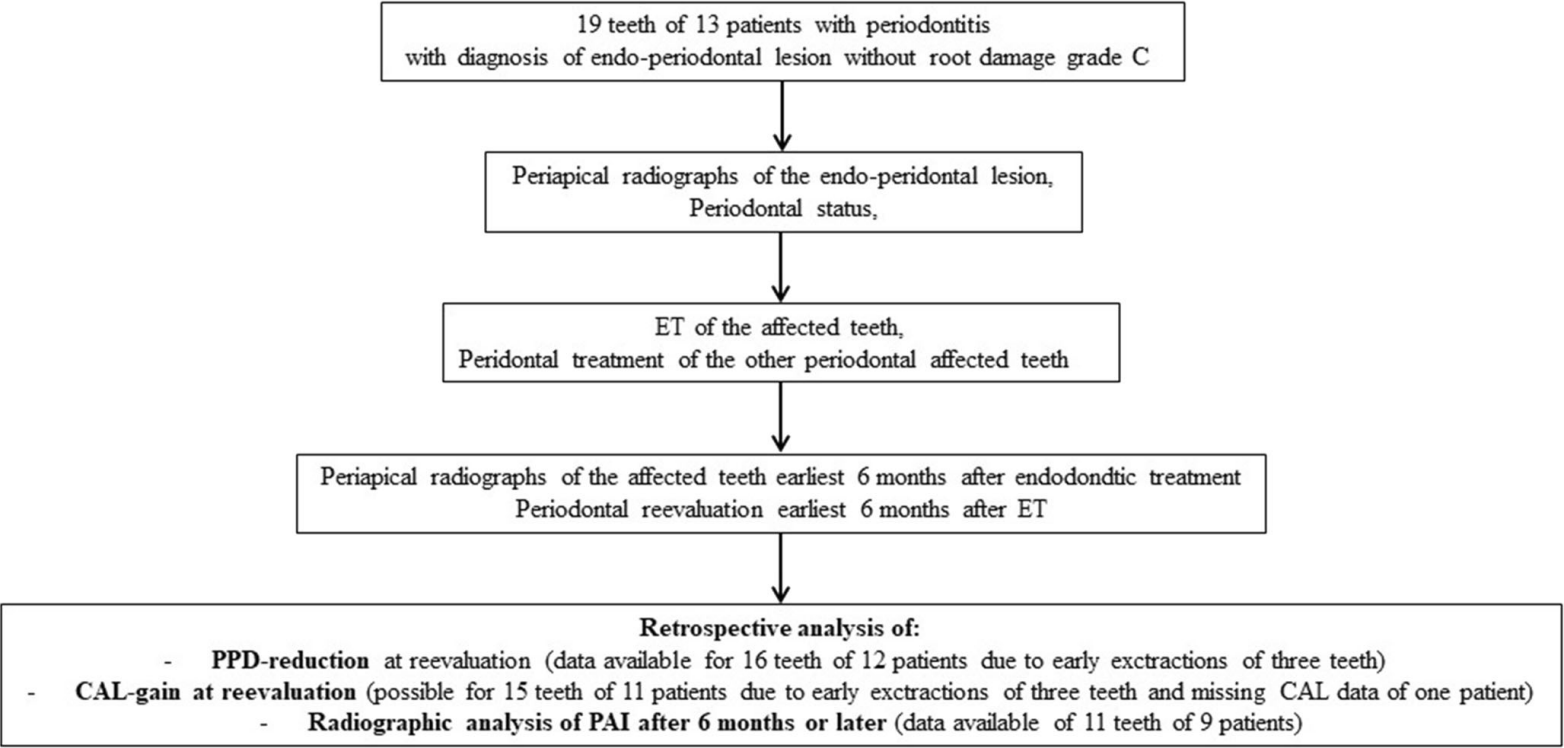
Flow chart of the study

For statistical analysis, the software SAS (SAS Institute, Cary, NC) v9.4 was used. For PPD and CAL gain, mean, standard deviation, median, minimum, and maximum were calculated. The Wilcoxon rank-sum test was used to assess differences between the means of baseline and reevaluation by means of calculating descriptive *p* values. Relative and absolute frequencies were calculated together with 95% Wilson-type rate confidence intervals for the change of PAI and the number of teeth classified as successful or failure. *p* values smaller than 0.05 were regarded as statistically significant. Due to the exploratory character of the trial, all *p* values are only to be interpreted descriptively. A study flow chart is shown in Fig. 2.

## Results

### Clinical results and quality of root canal fillings

Nineteen teeth of 13 patients were initially included. Seven were single-rooted teeth and 12 were multi-rooted teeth. Six single-rooted teeth were located in the lower jaw and one in the upper jaw; seven multi-rooted teeth were upper ones and 5 lower ones (Table 3).

**Table 3:** Patient data, teeth data, and PAI scores. *N* no, *Y* yes, *f* female, *m* male

ID	Sex	Age	Medical history	Smoker	Treated teeth (FDI)	Sensibility	Restoration	Extraction	Antibiotics within fmd	Clinical observation time (months)	PAI start	PAI evaluation	PAI improvement	Time to PAI evaluation (months)	PPD improvement	CAL improvement
1	f	48	Gastritis	0	42	1	No restoration	0	1	24	3	2	Successful	10	Failure	Failure
2	f	68	Healthy	0	24	1	Resin bonded filling	1	0	7	3	5	Failure	13	Failure	Failure
3	m	42	Asthma, diabetes	1	15	0	Resin bonded filling	1	1	56					Failure	Failure
4	m	44	Healthy	0	17	1	Resin bonded filling	1	0	80					Failure	Failure
5	m	34	Healthy	1	16	1	Bridge anchor	1	0	28					Failure	Failure
				1	46	1	Only	1		28	3	3	Failure	7	Failure	Failure
6	m	54	Depression	1	43	1	No restoration	0	0	13	3	1	Successful	9	Successful	Failure
7	f	43	Asthma, diabetes, hypertony	0	42	0	No restoration	1	0	49					Failure	Failure
8	m	43	Healthy	1	16	1	No restoration	0	0	6	1	3	Failure	6	Successful	Failure
				1	26	1	No restoration	0		6	4	4	Failure	8	Successful	Failure
9	m	68	Healthy	0	34	0	No restoration	0	1	83	5	2	Successful	14	Successful	Successful
10	m	22	Healthy	0	16	1	Carries	0	1	54					Successful	Failure
				0	26	1	No restoration	0		48	5	1	Successful	6	Failure	Failure
11	f	54	Gastritis, depression	0	24	0	Amalgam filling	0	0	40						
				0	45	1	Crown	0		37					Successful	Successful
				0	44	1	Crown	0		37					Successful	Successful
12	m	54	Healthy	0	36	1	No restoration	0	0	6	1	1	Failure	6		
				0	46	1	Resin bonded filling	0		11	2	2	Failure	11		
13	f	32	Healthy	0	36	0	Amalgam filling	0	0	74	5	1	Successful	17	Successful	Successful

The mean clinical observation period was 7.4 months. Three teeth were excluded due to early extraction before reevaluation after endodontic treatment. They were rated as a failure. Two of them were upper right molars and one of them a lower right molar. Therefore, 16 teeth in 12 patients were analyzed regarding the reduction of PPD as the main outcome variable. The quality of root canal fillings was overall rated as good. Only one tooth was rated short at length (Table 4) [11]. One tooth could not be rated as root canal filling was not performed because of extraction due to an abscess. There was a mean reduction of PPD of 1.34 ± 1.52 mm for the whole dentition (*p* = 0.002 for Wilcoxon test). Reduction ranged from − 1.00 to 5.00 mm with a median of 1.00 mm. Regarding the reduction only of the deepest pocket, a mean reduction of 3.19 ± 3.41 mm was observed (*p* = 0.002 for Wilcoxon test). It ranged from − 2.00 to 9.00 mm and the median was 3.5 mm.

**Table 4:** Quality of root canal fillings: rating according to Farzaneh et al. 2004 [11] (Table 1)

ID	Tooth	Root	Length	Density
1	42		Adequate	Good
2	24		Adequate	Good
3	15		Adequate	Good
4	17	No filling		
5	46	m	Adequate	Good
		d	Adequate	Good
6	43		Adequate	Good
7	42		Adequate	Good
8	16	mb	Adequate	Good
		p	Adequate	Good
		dp	Adequate	Good
	26	mb	Adequate	Good
		p	Adequate	Good
		dp	Adequate	Good
9	34		Adequate	Good
10	16	mb	Adequate	Good
		p	Adequate	Good
		db	Adequate	Good
	26	mb	Adequate	Good
		p	Adequate	Good
		db	Adequate	Good
11	24		Adequate	Good
	45		Adequate	Good
	44		Adequate	Good
12	36	m	Adequate	Good
		d	Adequate	Good
	46	m	Short	Good
		d	Adequate	Good
13	36	m	Adequate	Good
		d	Adequate	Good

*m* mesial, *d* distal, *b* buccal, *p* palatinal, *mb* mesiobuccal, *db* distobuccal

Data of CAL gain was available for 15 teeth in 11 patients. The mean CAL gain was 0.17 ± 3.62 mm for the whole dentition (*p* = 0.346 for Wilcoxon test). Gain ranged from − 10.00 to 4.00 mm with a median of 1.00 mm. Glancing at the sites with the deepest pocket, the mean CAL gain was 2.33 ± 3.75 mm (*p* = 0.033 for Wilcoxon test). It ranged from − 5.00 to 8.00 mm and the median was 3.00 mm.

Eight teeth (50%, Wilson-type 95% CI = [28.0%; 72.0%]) underwent a PPD reduction of ≥ 50% and were classified as success. With regard to the CAL gain, only four teeth (27%, Wilson-type 95% CI = [10.9%; 52.0%]) could be considered successful, showing a CAL gain of ≥ 50%. All four teeth were also successful concerning PPD reduction.

The presence of restorations, caries, smoking status, or antibiotic therapy showed no significant influence on the reduction of PPD.

### Results of radiographic evaluation by PAI

In total, 11 teeth in 9 patients were analyzed radiographically. Three of the teeth were single-rooted teeth and eight of them were multi-rooted teeth.

The mean observation time was 10 months. It ranged from 6 to 17 months. Five teeth (45.4%, Wilson-type 95% CI = [21.3%; 72.0%]) showed an improvement of PAI. Four were located in the lower jaw and one was located in the upper jaw. Three of them were single-rooted teeth and two of them multi-rooted teeth. One of them was an upper molar and one of them a lower molar. The single-rooted teeth were all mandibular.

Four teeth (36.4%, Wilson-type 95% CI = [15.2%; 64.6%]) showed no improvement and the periapical situation remained in the same state as before the treatment. Out of these, one was located in the upper jaw and three of them in the lower jaw. All of them were multi-rooted teeth.

Two teeth (18.2%, Wilson-type 95% CI = [5.1%; 47.7%]) showed an impairment of PAI (Table 2). Both were multi-rooted teeth and were located in the upper jaw. Overall, five teeth (45.4%, Wilson-type 95% CI = [21.3%; 72.0%]) could be classified as successful for showing an improvement of PAI score.

## Discussion

This retrospective study showed heterogenous results concerning the outcome of sole endodontic treatment of combined endo-periodontal lesions without root damage grade 3 of periodontally compromised patients. To the authors’ knowledge, this is the first study analyzing this aspect. There have been several case reports demonstrating different approaches to treating these lesions [15–19]. Some showed success but only in a few patients and most of them referred only to single-rooted teeth. In our study, more multi-rooted teeth were lost.

### Clinical situation

Within the observation period, statistically significant changes in means of PPD reduction were observed. The results failed to show statistical significance for overall CAL gain. Nevertheless, the deepest pockets managed to show a statistically significant improvement for PPD reduction as well as CAL gain. Concerning PPD reduction of 50% or more, 50% of the analyzed teeth were successfully treated, whereas for CAL gain, only 25% were considered successfully treated. This might be dependent on the size of the defect and the severity of general periodontal status, the dysbiotic status of the patient, and the status of the patient’s immune system. The periodontal destruction might be too severe to be handled only by root canal treatment. Studies already show that there is a similarity between the two different floras, but that the periodontal microflora is much more complex than the endodontic flora [3]. These patients might have benefited from additional root scaling or even open flap debridement and regenerative interventions [18]. In the study of Pico-Blanco et al., only single-rooted teeth were examined. Evidence shows that regenerative procedures at multi-rooted teeth are difficult and not very predictable [20]. In our study, only multi-rooted teeth were lost, suggesting that single-rooted teeth might benefit from sole endodontic treatment, and thus benefit from less interventive procedures. However, more and larger studies are required to investigate this assumption.

All successfully treated teeth classified by CAL gain were also classified as success by PPD reduction. However, not all successfully treated teeth concerning PPD reduction were a success regarding CAL gain. This shows that, for almost 50% of cases where periodontal healing occurred, the defect was resolved by the development of a recession rather than by healing through periodontal regeneration.

### Radiographic situation

The radiographic evaluation also showed heterogenous results, despite the change in the PAI score. It is interesting to note that impairment in PAI score was shown only at multi-rooted teeth of the upper jaw (two teeth). This might be due to the fact that multi-rooted teeth are harder to treat endodontically, and the anatomy of the teeth provides more niches for bacteria to hide and accumulate. Therefore, it might also be unsuccessful to treat multi-rooted teeth with scaling and root planning additionally as mentioned before [20]. Moreover, these teeth also show a worse prognosis at endodontic treatment in general [21].

The teeth with no changes in PAI score, meaning neither improvement nor impairment, were all multi-rooted teeth (three teeth). This is in line with our previous assumptions. Nevertheless, improvement in PAI could be detected in three multi-rooted teeth in addition to three single-rooted teeth.

Two of the three multi-rooted teeth were upper molars in one patient, and the other one a lower molar. The two patients were both non-smokers and both healthy and young. One of them was female and 33 years old, and the other one male and 22 years old. There are probably more influencing factors than the number of roots. However, more studies with larger cohorts are needed to provide conclusive results.

### Limitations

The retrospective design of the study should be considered a limitation. The small number of teeth is a further limitation. The study cohort was not large enough to be able to analyze the impact of general health factors statistically. In larger cohorts, the impact of health factors such as diabetes could have been respected in a statistical analysis. Those factors are known to be associated with periodontal breakdown [22, 23]. Although factors such as smoking, restoration, and caries decay have been statistically investigated, one should be cautious to reason that these factors do not influence the outcome because of the small number of teeth included in this study.

Moreover, the functional status of the teeth treated cannot be analyzed retrospectively. The treated teeth may had been functionally overloaded which might have had an impact on treatment outcome.

Although radiographically all root canal fillings appeared to be good at most sites, treatment was not performed by the same practitioner. This could be regarded as another bias to the results. Studies are available investigating the effect on the outcome of an endodontic treatment dependent on the education of the performing dentist [24].

In conclusion, this study was the first to investigate the treatment of sole endodontic treatment of teeth categorized by the new periodontal classification system. This study failed to prove sole endodontic treatment as a highly predictable therapy for teeth affected by those lesions. However, one should not forget that even eight of the teeth with a poor prognosis included in the study showed improvements of 50% or more concerning PPD reduction, and five teeth an improvement in PAI score. More studies with larger patient cohorts and a prospective character are needed to strengthen the evidence on modifying factors leading to success or failure of sole endodontic treatment. Further studies are required to evaluate treatment procedures and to establish a highly predictable treatment protocol involving endodontic and periodontal treatment approaches.
